# British Home for Incurables, Clapham

**Published:** 1893-05-13

**Authors:** 


### BRITISH HOME FOR INCURABLES, CLAPHAM, S.W.

The thirty-second annual meeting of the governors and subscribers of this charity was held at the Cannon Street Hotel on Thursday, May 4th, Earl Amhurst presiding. Amongst those present were Mr. Hale, General Elliott, Major Dundas, Mr. Payne, Mr. Baber, and others. Lord Amhurst, in moving the adoption of the report, said that subscriptions were much needed to carry on the work of the institution. The report was to be considered on the whole as satisfactory, though there had been a decrease of £302 5s. in the annual subscriptions as compared with last year. The donations which resulted from the annual dinner, and the garden party held at the laying of the foundation-stone of the new Home at Streatham, amounted to a considerable sum. The Home is not expected to be ready for occupation until next year, and the board have therefore arranged to keep on their present premises for another six months. The board are anxious to impress upon those desirous of benefiting the home by a legacy, the necessity of including the words "British Home for Incurables," in the form of bequest, as more than one such legacy has been lost to the institution by insufficient identification. The usual votes of thanks having been recorded, the election of candidates was proceeded with, and the evils of the canvassing system were abundantly shown by the unseemly bargaining for votes, which is the rule when admittance to a charity is obtained by such means.

